# The effect of noise-induced variance on parameter recovery from reaction times

**DOI:** 10.1186/s12859-016-0993-x

**Published:** 2016-03-31

**Authors:** Miguel A. Vadillo, Pablo Garaizar

**Affiliations:** Primary Care and Public Health Sciences, King’s College London, Addison House, Guy’s Campus, London, SE1 1UL UK; Division of Psychology and Language Sciences, University College London, 26 Bedford Way, London, WC1H 0AH UK; Faculty of Engineering, Universidad de Deusto, Avda. Universidades 24, Bilbao, 48007 Spain

**Keywords:** ex-Gaussian distribution, Internet-based experiments, Model fitting, Psychological experiments, Ratcliff Diffusion Model, Reaction times

## Abstract

**Background:**

Technical noise can compromise the precision and accuracy of the reaction times collected in psychological experiments, especially in the case of Internet-based studies. Although this noise seems to have only a small impact on traditional statistical analyses, its effects on model fit to reaction-time distributions remains unexplored.

**Results:**

Across four simulations we study the impact of technical noise on parameter recovery from data generated from an ex-Gaussian distribution and from a Ratcliff Diffusion Model. Our results suggest that the impact of noise-induced variance tends to be limited to specific parameters and conditions.

**Conclusions:**

Although we encourage researchers to adopt all measures to reduce the impact of noise on reaction-time experiments, we conclude that the typical amount of noise-induced variance found in these experiments does not pose substantial problems for statistical analyses based on model fitting.

## Background

Reaction times (RT) are probably the most extensively used dependent measure in behavioural and cognitive sciences [1, 2]. Many of the effects explored by researchers are typically in the range of just 30–100 milliseconds. To obtain an accurate estimation of such small effects, it is usually necessary to gather data from a relatively large number of observations and to use the most precise and accurate devices for the presentation of stimuli and the collection of RTs. Fortunately, thanks to the effort made by software developers, cognitive scientist have at their disposal a number of reliable software packages for the deployment of psychological experiments with strict temporal requirements [3–6].

Most of these packages were originally developed for running experiments in desktop computers and show very good performance in benchmarking studies [7, 8]. However, even using the best software and hardware, it is impossible to remove all sources of technical noise (e.g., timing constraints imposed by operating systems or input devices). Furthermore, with the increasing popularity of Internet-based experiments and the proliferation of general purpose libraries and frameworks for the design of online experiments [9, 10], researchers have expressed a logical concern about the accuracy and precision of web technologies [11, 12]. The studies that have addressed this issue so far have yielded promising results. During the last decade, several Internet-based experiments have successfully replicated well-known effects using RTs as their main dependent variable [13–17]. Furthermore, the results of simulation studies suggest that the amount of technical noise typically introduced by web technologies (and by computer input devices in general) has only a minor effect in the typical statistical comparisons made in experiments [18, 19].

In general, previous studies confirm that there is little reason to be suspicious about the accuracy and precision of the RTs collected in psychological experiments, even when they are conducted over the Internet. However, it is important to note that most of these studies have been based on rather simple approaches to data analyses that might reduce the impact of technical noise. Traditionally, before analysing RTs, researchers clean the data following some semi-standard procedures (e.g., trimming all RTs below or above a specific threshold or removing RTs more than 3 standard deviations away from the participant’s mean) and reduce the distribution of RTs to a single point estimate (e.g., averaging all valid RTs or computing their median) per participant and condition [20]. This procedure might ameliorate the impact of noise-induced variance because the data points most affected by it are likely to be either filtered or averaged out. However, an important shortcoming of this approach is that it neglects the rich information conveyed by the distribution of RTs, reducing it to a single point estimate.

Because of this, cognitive scientists are starting to replace these simple data-analysis strategies by more sophisticated alternatives that preserve more information from the RTs collected in psychological experiments. For example, fitting a model to the distribution of RTs provided by each participant is becoming an increasingly popular approach [21–26]. Instead of reducing all RTs to a single point estimate, the goal of these methods is to find out the properties or parameters of the distribution that best summarize the RTs provided by each participant. For instance, an experimental manipulation might not have an effect on the average RT, but it might increase or decrease the variability of RTs across participants. If researchers are interested in this effect, they can fit a model to the distribution of RTs provided by participants to find out whether the best-fitting value of the parameter(s) defining the variance in RTs has been affected by the experimental manipulation.

Although this data-analysis approach offers many advantages over traditional methods, we still do not know how the typical amount of technical noise introduced by operating systems, input devices, and web technologies affects model fitting. In the present study we explore the negative effect of noise-induced variance on parameter recovery using two popular models for the analysis of RTs. Simulations 1–3 explore to what extent the parameters recovered when fitting an ex-Gaussian distribution to RTs are deteriorated when the data set is affected by technical noise. Simulation 4 explores the same problem in a Ratliff Diffusion Model.

## Simulation 1

**Fig. 1:**
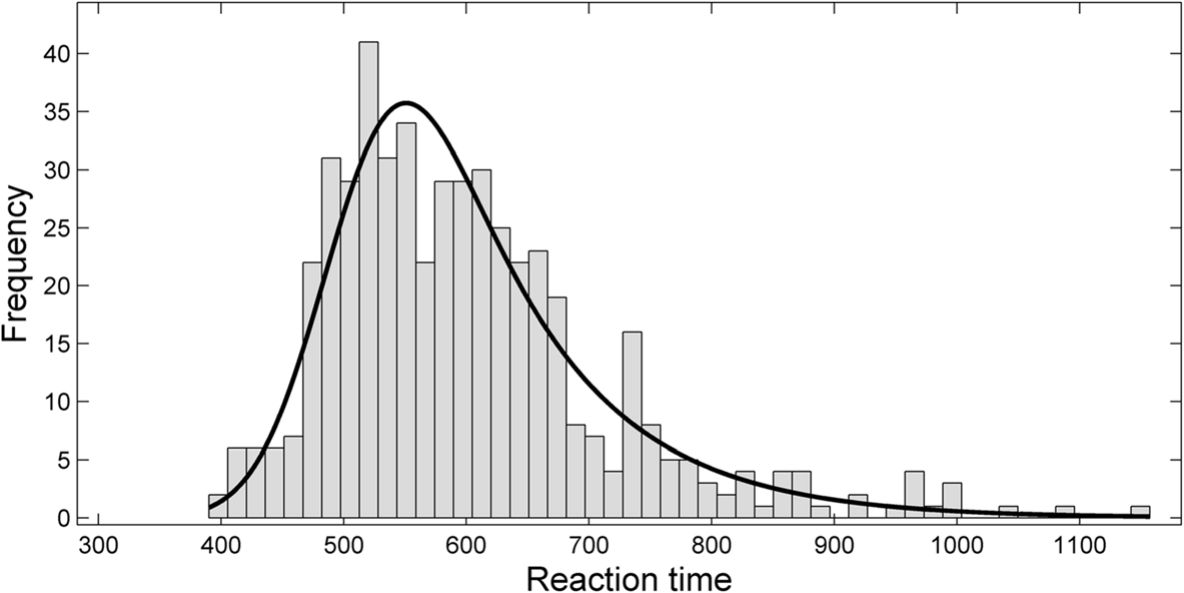
Histogram showing a fictitious data set following an ex-Gaussian distribution. The black line represents the best-fitting ex-Gaussian function

The ex-Gaussian distribution is probably the simplest and most popular model for reaction time data in psychological experiments. Figure 1 shows an example of an ex-Gaussian density function fitted to a fictitious data set. This distribution matches quite well the typical pattern of results found in cognitive psychology experiments, where most RTs are relatively fast but a few of them are consistently slow, giving rise to a highly skewed distribution that differs substantially from the normal distribution [20, 22, 27]. The ex-Gaussian distribution is the convolution of a normal and an exponential distribution. The three parameters of the ex-Gaussian distribution are the mean of the normal component (μ), the variance of the normal component (σ) and the mean of the exponential distribution (τ). The μ, σ, and τ parameters used for the example in Fig. 1 were 500, 50, and 100, respectively.

The goal of Simulation 1 was to assess how noise-induced variance would affect the recovery of the three parameters of the ex-Gaussian distribution. In each iteration, we sampled random data points from an ex-Gaussian distribution using random parameter values. Subsequently, we tried to recover the parameters of the model from which the data had been sampled using a maximum-likelihood estimation algorithm. After fitting the model to the original data set, we introduced noise in the data by adding a random amount of noise to each RT and we tried to repeat the parameter-recovery process on this new data set. The random noise introduced in the data was intended to mimic the technical noise in RT data collected in psychological experiments [18]. This process allows us to measure to what extent the accuracy of parameter estimation decreases after the introduction of noise. Specifically, our analyses explored systematic differences between the parameters recovered from the noiseless dataset and the parameters recovered from the same dataset after introducing noise.

### Method

The simulation was conducted using the DISTRIB toolbox for MATLAB [27]. The scripts used in Simulations 1–4 are publicly available at https://osf.io/r9fya/. Eighty data points were sampled from an ex-Gaussian distribution for each iteration. These were intended to model the number of trials per condition in a typical psychological experiment. The parameters of the ex-Gaussian distribution used in each iteration were sampled randomly from uniform distributions: μ ~ U(450, 550), σ ~ U(25, 75), and τ ~ U(50, 150). Two different versions of the data set were constructed, one of them without noise and the other with noise. As in previous studies [18], we simulated technical variance by adding a random value from a U(10, 100) distribution to each of the 80 data points. Then, we tried to retrieve the veridical parameters of the distribution from which the data set had been sampled originally. This was done separately for each version of the data set (with or without noise). The best-fitting parameters of the ex-Gaussian distribution were found using the egfit function from the DISTRIB toolbox, which relies on a maximum likelihood estimation procedure. The whole process was repeated 500 times with random parameter values and random noise in each iteration.

### Results and discussion

**Fig. 2:**
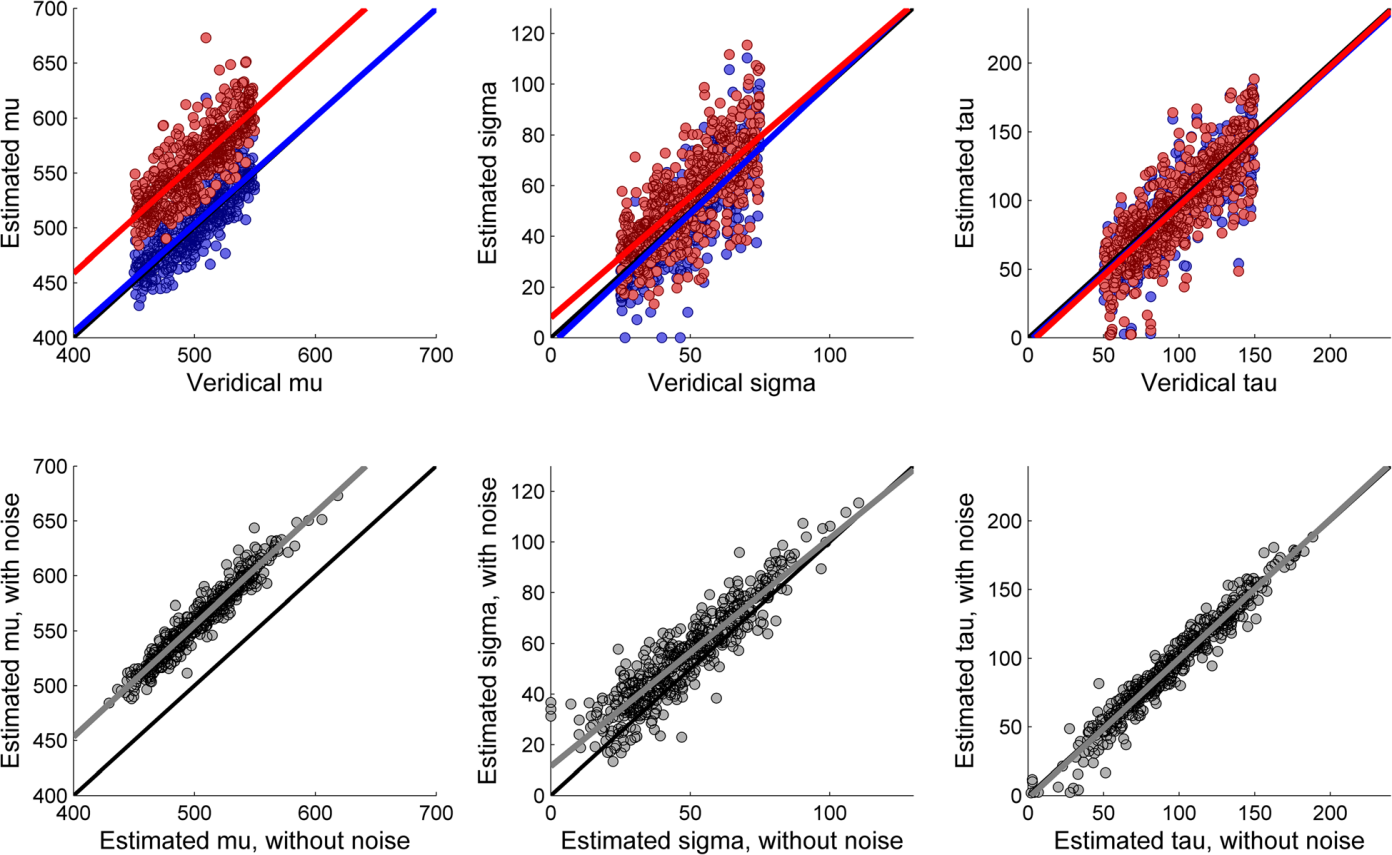
Results of Simulation 1

The results of the simulation are shown in Fig. 2. In the upper row, blue circles denote the parameters recovered in the data sets without noise and red circles denote parameters recovered from the data sets with random noise. The blue and the red lines represent the regression of lines of the relationship between the veridical values of the parameters and their retrieved values. For orientation purposes, a black line denotes the diagonal line. As can be seen, the blue regression lines overlap almost perfectly with the black diagonal, showing that in the case of noiseless data points there was a clear correspondence between the veridical parameters and the retrieved parameters. Note however the generous amount of variation around the regression line revealing that the accuracy of model-fitting was less than perfect. The correspondence between veridical and retrieved parameters was also reasonably good for data sets with noise (red line), although the inclusion of noise induced some systematic biases in the retrieved parameters.

The effects of noise-induced variance are clearer in the lower row of Fig. 2, showing the relationship between the parameters recovered in the noiseless dataset and the parameters recovered after adding noise to those datasets. As in the upper row, the black line denotes the diagonal line and the grey line denotes the best-fitting regression. The coefficients (intercept and slope) of the regressions for each parameter (μ, σ, and τ) are shown in Table 1, along with their 95 % confidence intervals (CI). As can be seen, the estimated μ tends to be systematically larger for the datasets with noise than for the datasets without noise. However, the slope of the regression is not significantly different from one, suggesting that the difference between the retrieved parameters remains similar across different values of μ. In the case of the estimated σ, the intercept is significantly larger than zero, but the slope is slightly lower than one. This suggests that the estimated σ tends to be larger in the datasets with noise-induced variance, but more so in the case of datasets with small σ. Visual inspection suggests that the inclusion of noise had a somewhat smaller effect on the estimated τ. This impression is confirmed by the fact that the intercept is not significantly different from zero and the slope is not significantly different from one.

**Table 1:** Results of Simulation 1

	μ			σ			τ		
	*β*	95 % CI	*p*-value	*β*	95 % CI	*p*-value	*β*	95 % CI	*p*-value
Intercept	55.92	[44.45, 67.39]*	< .001	14.01	[12.13, 15.90]*	< .001	0.74	[−1.38, 2.87]	.490
Slope	0.99	[0.97, 1.02]	.916	0.85	[0.81, 0.89]*	< .001	0.98	[0.96, 1.01]	.246

Note. coefficients of the regressions shown in the second row of Fig. 2. Intercepts are marked with an asterisk with the 95 % confidence interval (CI) of the regression coefficient excludes zero. Slopes are considered statistically significant when the CI of the regression coefficient excludes 1

The results of the simulation reveal that noise-induced variance introduces some systematic biases in the fit of the ex-Gaussian distribution to RT data. As could be expected, the estimated value of μ gets larger, given that the whole distribution of RTs is delayed by the addition of random noise. Noise also biases the estimation of σ, particularly for low values of σ. In contrast, the estimation of τ seems to remain unaffected by noise-induced variance, at least within the parameters of the present simulation.

## Simulation 2

In Simulation 1 we modelled technical noise by adding a random value from a U(10, 100) distribution to each RT. This strategy has been adopted in previous simulation studies [18] and, as explained in the Conclusions, it is a reasonable assumption given the technical constraints imposed by the hardware and software typically used in psychological research. However, this assumption might not be valid for all experimental setups. To address this concern, in Simulation 2 we manipulated the upper bound of the uniform distribution from where noise is sampled. Specifically, we replicated Simulation 1, but using a range of uniform distributions from U(10, 50) to U(10, 200).

### Methods

All the details of the design and procedure were identical to those of Simulation 1 except that the upper bound of the noise distribution was manipulated from 50 to 200 in steps of 10. Exploratory analyses suggested that a large number of iterations would be needed to obtain accurate estimations of the target regression coefficients. Consequently, 1000 iterations were conducted for each condition (instead of the 500 used in Simulation 1).

### Results and discussion

**Fig. 3:**
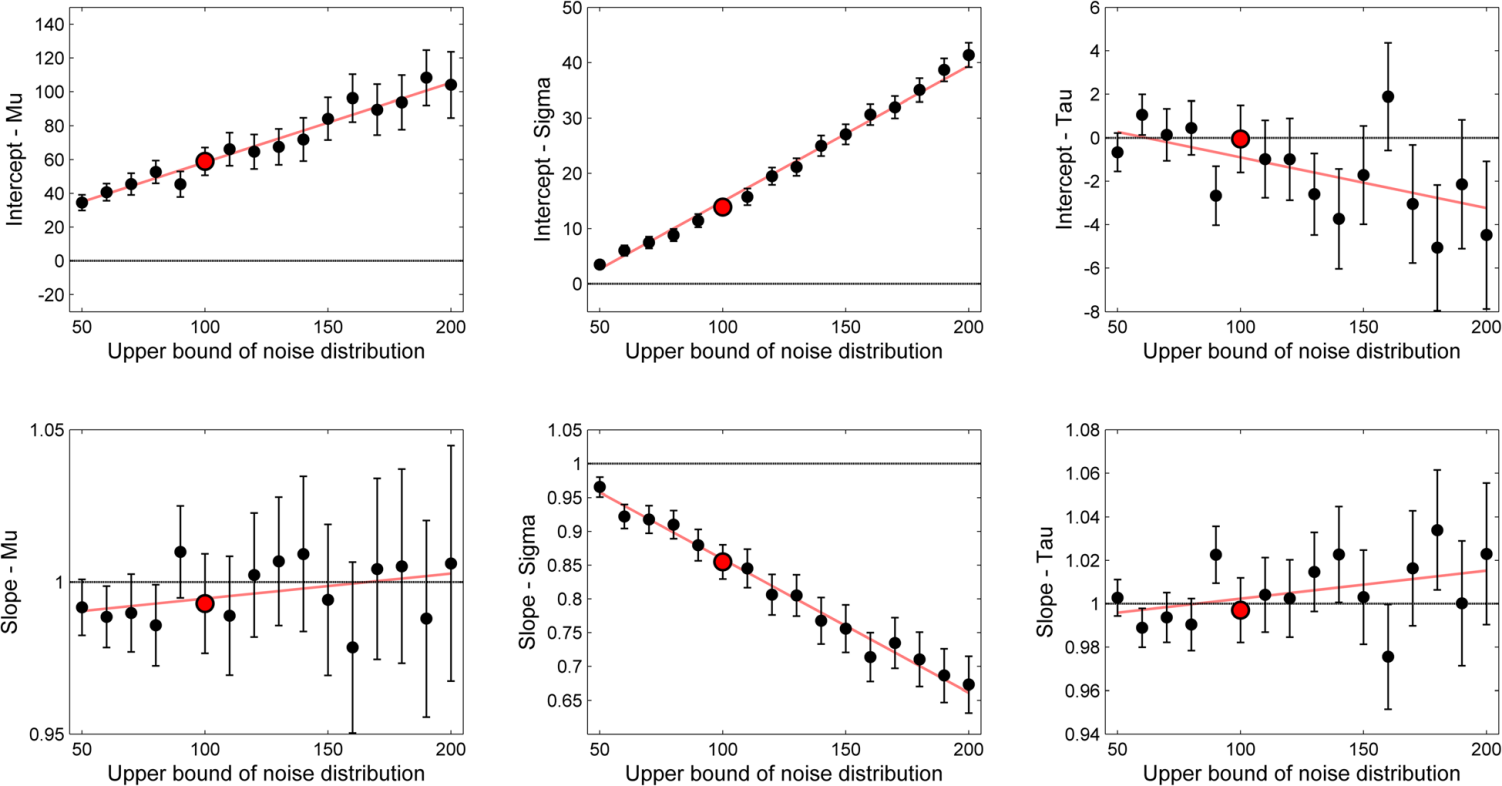
Results of Simulation 2. Error bars denote 95 % confidence intervals. The red line represents the best-fitting linear regression using a weighted least squares algorithm, where the weight of each data point is inversely proportional to the width of the confidence interval

As in Simulation 1, we conducted a linear regression predicting the estimated parameters in the datasets with noise from the estimated parameters in the datasets without noise. The resulting regression coefficients and their 95 % confidence intervals are reported in Fig. 3. These values are analogous to the results presented in Table 1 for Simulation 1. In fact, one of the conditions in Simulation 2 (marked in red in Fig. 3) was an exact replication of Simulation 1 and, unsurprisingly, yielded very similar results. Overall, Simulation 2 confirms that noise-induced variance biases the intercept of the estimated μ but not its slope. This means that the estimated μ becomes larger if substantial amounts of noise are included. However, this effect remains constant across different levels of μ. The impact of noise on the estimated σ is more complex. As can be seen in Fig. 3, noise affects both the intercept and the slope of the regression. This confirms the trend found in Simulation 1, where noise affected the estimation of small σ values, but not of large σ values. Simulation 2 shows that this trend becomes stronger with larger amounts of noise. Finally, and most interestingly, noise seemed to have very little effect on the intercept and slope of τ. Only a minority of the regression coefficients were significantly different from the critical values (zero or one) and this only happened for wide noise distributions that included very large values. Overall, this suggests that the lack of biases in the estimated τ that we found in Simulation 1 hold across very different noise distributions. It is also interesting to point out that, overall, the confidence intervals of both intercepts and slopes become wider with larger amounts of varying noise, particularly for μ and τ. This may suggest that in a given experiment, relatively constant noise may have less impact on the variation of the estimated parameters.

## Simulation 3

In Simulation 3 we explored to what extent the addition of noise might make it more difficult to find significant differences in the recovered parameters across experimental conditions.

### Methods

A different simulation was conducted for each parameter of the ex-Gaussian distribution and for 5 different effect sizes. In each case, we first generated data from 500 fictitious participants using the same methods as in Simulation 1. For each participant, we first chose random parameters from uniform distributions, μ ~ U(450, 550), σ ~ U(25, 75), and τ ~ U(50, 150), and then we extracted 80 data points from an ex-Gaussian distribution with those parameters. These 80 data points represented the control condition for that participant.

To generate an experimental condition, we added an “effect size” to one of the parameters and we sampled 80 additional data points from the new ex-Gaussian distribution. Specifically, we tested five different effect sizes: 10, 20, 30, 40, and 50 msecs. For instance, if for one participant μ = 500 in the control condition, then her data for the experimental condition was sampled from a distribution with μ = 510 (or 520, 530, 540, and 550, to simulate effect sizes 10, 20, 30, 40, and 50, respectively). To quantify the difference between both conditions, control and experimental, we computed a paired-samples *t*-test with data from the 500 participants of each simulation and we converted the *t* value to a Cohen’s *d* effect size estimate.

For each participant, we created two versions of the control and the experimental condition, one of them without noise and one with noise. Noise was added using the same procedure as in Simulations 1 and 2: A random value sampled from a U(10, 100) distribution was added to each data point.

### Results and discussion

The results of Simulation 3 are summarized in Table 2. The crucial question was whether the *d* values of the comparisons between the experimental and control conditions were lower for the data sets with noise. As can be seen in Table 1 this seemed to be the case for all comparisons. In all cases, comparing the RTs in the control and experimental conditions yielded larger *d*’s for data sets without noise than for data sets with noise. For instance, within the parameters of our simulations, when data were not affected by noise an increase of 50 ms in parameter μ gave rise to a difference of *d* = 2.18 between the estimated μ of the experimental condition and the estimated μ of the control condition. However, when noise was added the size of the difference dropped to 1.88. The same pattern is observed across all effect sizes and parameters. This drop in effect size has obvious practical implications. In addition to Cohen’s *d*, each cell in Table 2 includes the minimum number of participants that should be included to achieve a 80 % statistical power to detect the experimental manipulation given the effect sizes denoted by *d* in a within-subjects experiment with α = .05. These sample sizes were computed using G*Power 3 [28]. As can be seen, a non-trivial increase in sample size is necessary to compensate for the lower effect sizes found in data sets with noise, particularly in the first two rows of the table, where effect sizes are in line with the range of values typically found in behavioural studies.

**Table 2:** Results of Simulation 3

	μ		σ		τ	
Effect size	No noise	Noise	No noise	Noise	No noise	Noise
10 ms	*d* = 0.31	*d* = 0.30	*d* = 0.53	*d* = 0.48	*d* = 0.40	*d* = 0.35
	*n*_80%_ = 84	*n*_80%_ = 90	*n*_80%_ = 30	*n*_80%_ = 37	*n*_80%_ = 52	*n*_80%_ = 67
20 ms	*d* = 0.90	*d* = 0.77	*d* = 1.13	*d* = 0.96	*d* = 0.67	*d* = 0.61
	*n*_80%_ = 12	*n*_80%_ = 16	*n*_80%_ = 9	*n*_80%_ = 11	*n*_80%_ = 20	*n*_80%_ = 24
30 ms	*d* = 1.37	*d* = 1.18	*d* = 1.57	*d* = 1.31	*d* = 1.04	*d* = 0.99
	*n*_80%_ = 7	*n*_80%_ = 8	*n*_80%_ = 6	*n*_80%_ = 7	*n*_80%_ = 10	*n*_80%_ = 11
40 ms	*d* = 1.42	*d* = 1.27	*d* = 1.85	*d* = 1.65	*d* = 1.36	*d* = 1.26
	*n*_80%_ = 7	*n*_80%_ = 8	*n*_80%_ = 5	*n*_80%_ = 6	*n*_80%_ = 7	*n*_80%_ = 8
50 ms	*d* = 2.18	*d* = 1.88	*d* = 2.40	*d* = 2.11	*d* = 1.44	*d* = 1.36
	*n*_80%_ = 4	*n*_80%_ = 5	*n*_80%_ = 4	*n*_80%_ = 5	*n*_80%_ = 6	*n*_80%_ = 7

Note. Cohen’s *d* estimates for the size of the comparison between the control condition and the experimental condition. *n*_80%_ denotes the number of participants that would be needed to achieve 80 % statistical power in a two-tailed *t*-test for related samples, given the effect size denoted by *d* and an α of .05

## Simulation 4

**Fig. 4:**
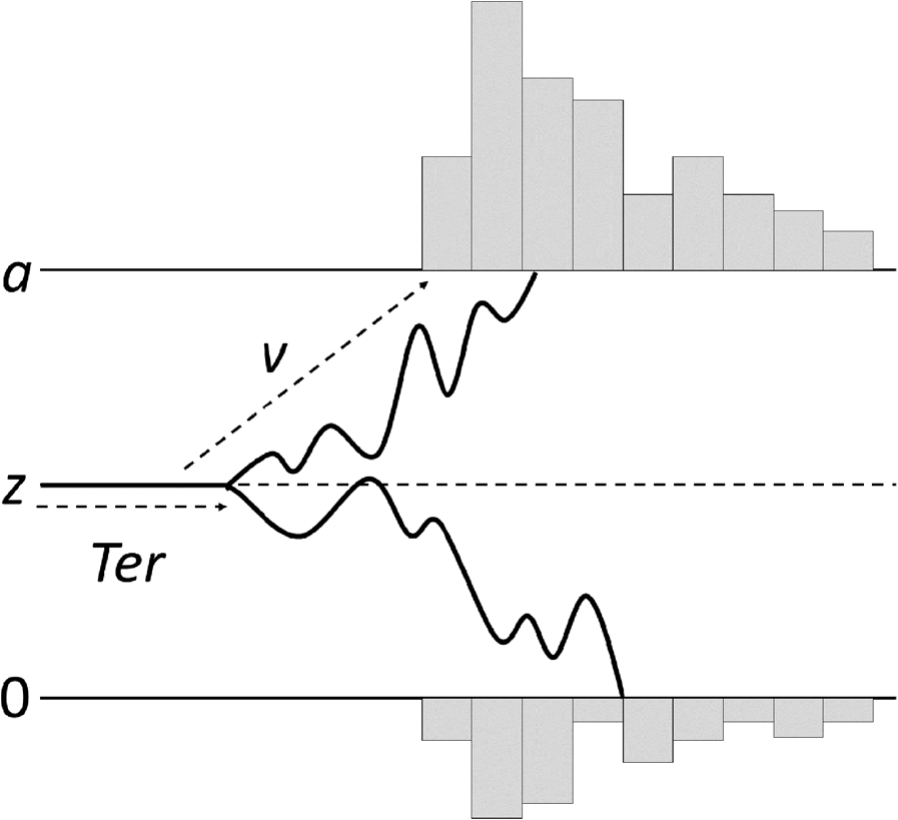
Graphical representation of the Ratcliff Difussion Model

Although the simplicity of the ex-Gaussian distribution and its relatively good fit to empirical data make it an appealing model for RT data, over the last years other models have gained popularity among cognitive scientists. The Ratcliff diffusion model (RDM), the linear ballistic accumulator and the leaky competing accumulator are good examples of the new trend towards more sophisticated RT models [21, 23, 24]. The RDM is possibly the most influential among them. The RDM is a formal theory of the processes involved in 2-alternative forced-choice tasks [25, 26, 29]. According to this model, selecting and executing the appropriate response to a given stimulus requires a noisy accumulation of evidence over time. Figure 4 represents an example of this process. The model assumes that the participant decides to perform a response when the accumulation of evidence reaches a threshold, denoted by parameter *a* in Fig. 4. Other things being equal, the lower the boundary *a*, the faster the responses. Although the accumulation of evidence is influenced by chance, there is a systematic component in the accumulation function that represents the general slope or drift rate of the function. This slope is represented by parameter *v* in Fig. 4. Smaller drift values result in slower RTs and in a less skewed distribution. Given the noise in the accumulation of evidence, the function can accidentally cross the lower threshold, 0, which would give rise to an incorrect response. This is more likely to happen when the drift is relatively reduced and the boundary separation is small. In some experimental paradigms, participants might have a bias towards a specific response. The model can capture this overall preference for a given response by changing the starting point of the accumulation function, denoted by *z*. Finally, the reaction times can be faster or slower for reasons that are not specifically considered in the model such as, for example, the time required to encode stimuli or to execute responses. All these extra-decisional components are captured by the parameter *Ter*. Changes in *Ter* result in slower reaction times for correct and incorrect responses, but they do not affect the shape of the distribution of reaction times.

At the time of writing this report, the number of citations of the seminal paper applying the RDM to RT data from psychological experiments [23] is well above 1400. Since the original publication of the model, the RDM has been applied to countless experimental paradigms, including lexical decision, implicit attitudes, or visual search [30–32]. Given the increasing popularity of the RDM, we decided to conduct a conceptual replication of Simulation 1 but using the RDM to sample data and to retrieve parameters, instead of the simpler ex-Gaussian distribution.

### Method

The general method was similar to the one used for Simulation 1. In each iteration, we first sampled 100 data points from a diffusion process using the DMAT toolbox for MATLAB [33]. The number of data points was increased from 80 to 100 because it is recommended to collect a relatively large number of reaction times per condition to fit the RDM. As in Simulation 1, the specific values of parameters *a*, *v* and *Ter* in each iteration were randomly sampled from a uniform U(0.15, 0.25) distribution. The value of *z* was set to *a*/2 in all simulations. Similarly, although the full RDM includes additional parameters to model across-trial variance in three of the main parameters (*η*, *s*_*z*_ and *s*_*t*_), all these parameters were set to zero in the present simulations. As in Simulation 1, we created two versions of the data set: A version without noise and a version with added noise that tried to mimic the technical variance introduced by the computer in the measurement of RTs. Noise was added following the same procedure as in Simulation 1. Finally, we tried to recover the original parameters of the RDM that were used to generate the data. The RDM was fitted to both data sets (with and without noise) using the multiestv4 function from the DMAT toolbox. During recovery, the value of *z* was constrained to be *a*/2, and the values of *η*, *s*_*z*_ and *s*_*t*_ were constrained to zero. As in Simulation 1, we conducted 500 simulations, each of them with different parameter values.

### Results and discussion

**Fig. 5:**
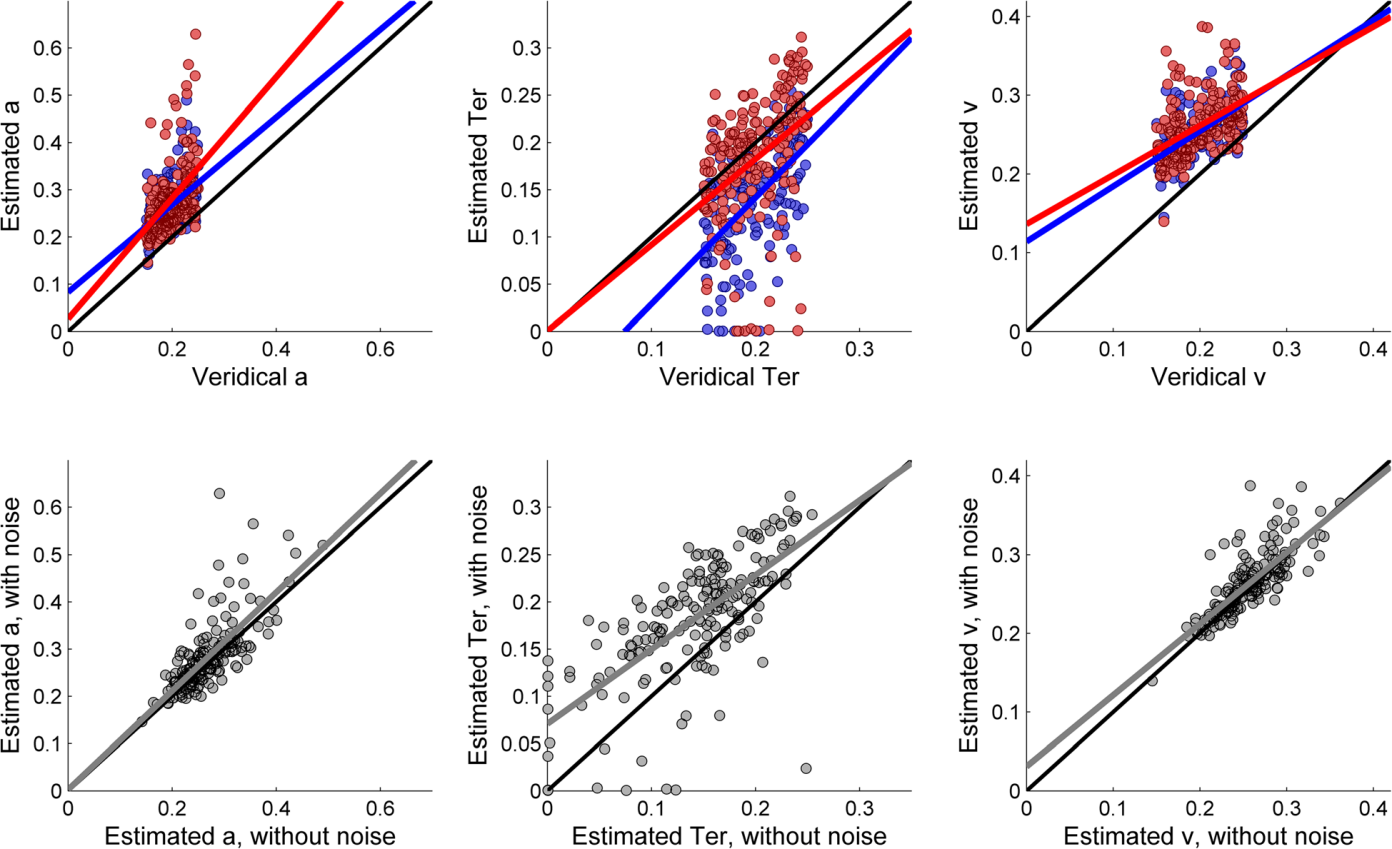
Results of Simulation 2

Figure 5 shows the results of the simulation. The upper row shows the correspondence between both sets of estimated parameters (with and without noise) and the veridical parameters. As in Simulation 1, the black line represents the theoretical regression line where all the data points should fall if the recovered parameters had been identical to the veridical parameters. As can be seen, even in data sets without noise, the recovered parameters differ substantially from the veridical parameters. Specifically, the boundary separation, *a*, and the drift rate, *v*, tended to be overestimated, while the non-decisional component, *Ter*, was systematically underestimated.

The lower row in Fig. 5 shows the relationship between the estimated parameters in the datasets without noise and the estimated parameters in the corresponding datasets with noise. These scatterplots suggest that, overall, the estimated *a* was relatively unaffected by noise. However, the estimated *v* and particularly the estimated *Ter* were slightly affected by noise. Table 3 shows the coefficients of the regressions plotted in the lower row of Fig. 5. The 95 % confidence intervals of the regressions coefficients confirm that the effects of noise were confined to the estimation of parameters *Ter* and *v*, with no significant effect on the estimation of *a*. For both *Ter* and *v*, the intercept of the regression is larger than zero and the slope is less than one, suggesting that in both cases, noise induces an overestimation of parameters with small values, but this bias is progressively reduced for larger values.

**Table 3:** Results of Simulation 4

	*a*			*Ter*			*v*		
	*β*	95 % CI	*p*-value	*β*	95 % CI	*p*-value	*β*	95 % CI	*p*-value
Intercept	0.0026	[−0.020, 0.025]	.823	0.0708	[0.060, 0.082]*	< .001	0.0309	[0.016, 0.046]*	< .001
Slope	1.0439	[0.961, 1.127]	.301	0.7881	[0.718, 0.859]*	< .001	0.9060	[0.848, 0.965]*	.002

Note. Unstandardized coefficients of the regressions shown in the second row of Fig. 5. Intercepts are marked with an asterisk with the 95 % confidence interval (CI) of the regression coefficient excludes zero. Slopes are considered statistically significant when the CI of the regression coefficient excludes 1

## Conclusions

The results of our simulations suggest that the amount of technical noise typically introduced by software or by input devices does have an effect on parameter-recovery when fitting a model to the distribution of RTs. However, these effects were usually confined to specific parameters. For instance, in the case of the ex-Gaussian distribution, the added noise gives rise to an increase in the mean of the normal component, μ, and it also biases the estimation of σ, especially when σ is low. However, noise has little or no effect on the estimation of τ. Similarly, in the case of the RDM, technical noise had an effect on *Ter* and *v*, but not on *a*. Simulation 3 showed that the effect of an experimental manipulation on the parameters of an ex-Gaussian distribution becomes smaller after adding some amount of technical noise. However, within the parameters of our simulation, the decline in effect sizes was relatively small and can be easily compensated using slightly larger sample sizes.

Of course, our conclusions are only valid to the extent that our procedure to model technical noise mirrors the actual sources of noise that affect psychological experiments. Although the procedure that we chose in Simulations 1 and 3, adding a random value from a U(10, 100) distribution, is typical in previous studies [18], it might not reflect accurately the distribution of technical noise under all circumstances. We suspect, however, that this a reasonable estimation of the amount of technical noise introduced by the experimental hardware and software. The delay introduced by keyboards has been extensively explored in multiple benchmarking studies with different results depending on the specific model. These studies show that the average delay rarely goes beyond 36 ms and is typically around 15–20 ms [34–39]. Similar delays (although perhaps more variable across brands and models) are observed in mice [37, 38, 40–42]. Although the main timing function of Windows, the so-called “wall clock”, is only updated every 15 msecs [43, 44], some timing functions in Windows can achieve time resolutions below the millisecond [45]. The accuracy of web applications depends on the accuracy of the timing function invoked, but under favourable conditions, the latest timing functions developed by the World Wide Web Consortium (W3C), such as the High Resolution Time API, have resolutions in the order of microseconds and minimal function call costs [46]. Unfortunately, this API is not used in all experiments and it is not supported by all web platforms yet. In light of this information, the assumption that technical noise introduces delays of 10–100 msecs might be rather pessimistic under many conditions. In any case, the results of Simulation 2 show that our conclusions hold even under even more negative (or positive) conditions. The interested reader can adapt the scripts of our simulations to explore the effects of noise under different assumptions.

The validity of our simulations also depends on the number of trials simulated per participant. We decided to simulate 80 trials per participant in Simulations 1–3 and 100 trials per participant in Simulation 4 because, compared to the ex-Gaussian distribution, the RDM requires a relatively large number of data points of get an accurate estimation of the parameters underlying the RT distribution [25, 26]. The number of trials needed to fit the RDM depends, among other factors, on the number of parameters of the model that are allowed to vary. In Simulation 4 we only allowed *a*, *Ter*, and *v* to vary. The results plotted in Fig. 5 suggest that the estimation of these three parameters with just 100 trials was less than perfect but, overall, well correlated with the veridical parameters. However, these results might not hold for data sets with fewer data points or for attempts to recover other parameters of the RDM (like *z*, or the variances of the parameters across trials). Although some experiments using the RDM collected RTs from 100 or fewer trials per condition [30, 47, 48], experiments involving more than 1000 trials are not infrequent [49, 50]. Again, we invite the readers to adapt our scripts if they wish to explore the effect of noise on larger or smaller datasets involving more or fewer free parameters.

Although our simulations suggest that the typical amount of technical noise is unlikely to cause a large bias in model fitting, nevertheless we would like to encourage researchers to make all efforts to minimize the sources of noise in their experiments. As recently put by Plant [11, 51], the variability in software and hardware used in experimental settings might account for the lack of replicability of some findings, especially in areas that rely on sophisticated paradigms and devices like EEG, MEG and fMRI. In the particular case of Internet experiments, not all technologies and time functions are equally accurate for the presentation of stimuli and the collection of reaction times [12, 17, 52–54]. Depending on the particular choices made by researchers, their experiments might contain more or less technical noise than the one implemented in our simulations.

## References

[1] Luce RD (1986). Response times.

[2] Posner MI (2005). Timing in the brain: mental chronometry as a tool in neuroscience. PLoS Biol.

[3] Forster KI, Forster JC (2003). DMDX: A Windows display program with millisecond accuracy. Behav Res Meth Ins C.

[4] Mathôt S, Schreij D, Theeuwes J (2011). OpenSesame: An open-source, graphical experiment builder for the social sciences. Behav Res Methods.

[5] Peirce JW (2007). PsychoPy: Psychophysics software in Python. J Neurosci Meth.

[6] Schneider W, Eschman A, Zuccolotto A (2002). E-Prime user’s guide.

[7] Garaizar P, Vadillo MA (2014). Accuracy and precision of visual stimulus timing in PsychoPy: No timing errors in standard usage. PLoS One.

[8] Garaizar P, Vadillo MA, López-de-Ipiña D, Matute H (2014). Measuring software timing errors in the presentation of visual stimuli in cognitive neuroscience experiments. PLoS One.

[9] de Leeuw JR (2014). jsPsych: A JavaScript library for creating behavioural experiments in a Web browser. Behav Res Methods.

[10] Schubert TW, Murteira C, Collins EC, Lopes D (2013). ScriptingRT: A software library for collecting response latencies in online studies of cognition. PLoS One.

[11] Plant RR. A reminder on millisecond timing accuracy and potential replication failure in computer-based psychology experiments: An open letter. Behav Res Methods. in press. 10.3758/s13428-015-0577-0 25761394

[12] Reimers S, Stewart N. Presentation and response timing accuracy in Adobe Flash and HTML5/JavaScript Web experiments. Behav Res Methods. in press. 10.3758/s13428-014-0471-1 PMC4427652 24903687

[13] Crump MJC, McDonnell JV, Gureckis TM (2013). Evaluating Amazon’s Mechanical Turk as a tool for experimental behavioral research. PLoS One.

[14] McGraw KO, Tew MD, Williams JE (2000). The integrity of web-delivered experiments: Can you trust the data?. Psychol Sci.

[15] Nosek BA, Banaji MR, Greenwald AG (2002). Harvesting implicit group attitudes and beliefs from a demonstration website. Group Dyn Theor Res.

[16] Reimers, Maylor EA (2005). Task switching across the life span: effects of age on general and specific costs. Dev Psychol.

[17] Reimers S, Stewart N (2007). Adobe Flash as a medium for online experimentation: a test of reaction time measurement capabilities. Behav Res Methods.

[18] Brand A, Bradley MT (2012). Assessing the effect of technical variance on the statistical outcomes of web experiments measuring response times. Soc Sci Comput Rev.

[19] Damian MF (2010). Does variability in human performance outweigh imprecision in response devices such as computer keyboards?. Behav Res Methods.

[20] Ratcliff R (1993). Methods for dealing with reaction time outliers. Psychol Bull.

[21] Donkin C, Brown S, Heathcote A (2011). Drawing conclusions from choice response time models: a tutorial using the linear ballistic accumulator. J Math Psychol.

[22] Heathcote A, Popiel SJ, Mewhort DJK (1991). Analysis of response time distributions: an example using the Stroop task. Psychol Bull.

[23] Ratcliff R (1978). A theory of memory retrieval. Psychol Rev.

[24] Usher M, McClelland JL (2001). The time course of perceptual choice: the leaky competing accumulator model. Psychol Rev.

[25] Voss A, Nagler M, Lerche V (2013). Diffusion models in experimental psychology. Exp Psychol.

[26] Wagenmakers EJ (2009). Methodological and empirical developments for the Ratcliff diffusion model of response times and accuracy. Eur J Cogn Psychol.

[27] Lacouture Y, Cousineau D (2008). How to use MATLAB to fit the ex-Gaussian and other probability functions to a distribution of response times. Tutor Quant Methods Psychol.

[28] Faul F, Erdfelder E, Lang AG, Buchner A (2007). G*Power 3: a flexible statistical power analysis program for the social, behavioral, and biomedical sciences. Behav Res Methods.

[29] Smith PL, Ratcliff R, Forstmann BU, Wagenmakers EJ (2015). An introduction to the diffusion model of decision making. An introduction to model-based cognitive neuroscience.

[30] Klauer KC, Voss A, Schmitz F, Teige-Mocigemba S (2007). Process components of the implicit association test: a diffusion-model analysis. J Pers Soc Psychol.

[31] Ratcliff R, Thapar A, Gomez P, McKoon G (2004). A diffusion model analysis of the effects of aging in the lexical-decision task. Psychol Aging.

[32] Tseng YC, Glaser JI, Caddigan E, Lleras A (2014). Modeling the effect of selection history of pop-out visual search. PLoS One.

[33] Vandekerckhove J, Tuerlinckx F (2008). Diffusion model analysis with MATLAB: A DMAT primer. Behav Res Methods.

[34] Graves R, Bradley R (1987). Millisecond interval timer and auditory reaction time programs for the IBM PC. Behav Res Meth Ins C.

[35] Forster JC (2007). DMDX updates page.

[36] Neath I, Earle A, Hallett D, Surprenant A (2011). Response time accuracy in Apple Macintosh computers. Behav Res Methods.

[37] Plant RR, Turner G (2009). Millisecond precision psychological research in a word of commodity computers: New hardware, new problems?. Behav Res Methods.

[38] Segalowitz S, Graves R (1990). Suitability of the IBM XT, AT, and PS/2 keyboard, mouse, and game port as response devices in reaction time paradigms. Behav Res Meth Ins C.

[39] Shimizu H (2002). Measuring keyboard response delays by comparing keyboard and joystick inputs. Behav Res Meth Ins C.

[40] Beringer D (1989). Touch panel sampling strategies and keypad performance comparisons. Proc Hum Factors Ergon Soc Annu Meet.

[41] Crosbie J (1990). The Microsoft mouse as a multipurpose response device for the IBM PC/XT/AT. Behav Res Meth Ins C.

[42] Plant R, Hammond N, Whitehouse T (2002). Toward an experimental timing standards lab: Benchmarking precision in the real world. Behav Res Meth Ins C.

[43] Nguyen K, Shankar V. Power analysis guide for Windows. Revision 1.0. https://software.intel.com/en-us/articles/power-analysis-guide-for-windows Accessed 24 May 2016.

[44] Russinovich M, Solomon D, Ionescu A. Windows® internals. Microsoft Press; Redmond, Washington, USA. 2009.

[45] Kuperberg M, Krogmann M, Reussner R (2009). TimerMeter: Quantifying properties of software timers for system analysis.

[46] Mann J (2012). High resolution time. W3C proposed recommendation.

[47] Germar M, Schlemmer A, Krug K, Voss A, Mojzisch A (2014). Social influence and perceptual decision making: a diffusion model analysis. Pers Soc Psychol B.

[48] Metin B, Roeyers H, Wiersema JR, van der Meere JJ, Thompson M, Sonuga-Barke E (2013). ADHD performance reflects inefficient but not impulsive information processing: a diffusion model analysis. Neuropsychology.

[49] Leite FP, Ratcliff R (2011). What cognitive processes drive response biases? A diffusion model analysis. Judgm Decis Mak.

[50] Ratcliff R, Thapar A, McKoon G (2006). Aging and individual differences in rapid two-choice decisions. Psychon B Rev.

[51] Plant RR, Quinlan PT (2013). Could millisecond timing errors in commonly used equipment be a cause of replication failure in some neuroscience studies?. Cogn Affect Behav Ne.

[52] Garaizar P, Vadillo MA, López-de-Ipiña D (2014). Presentation accuracy of the Web revisited: animation methods in the HTML5 era. PLoS One.

[53] Keller F, Gunasekharan S, Mayo N, Corley M (2009). Timing accuracy of web experiments: a case study using the Webexp software package. Behav Res Methods.

[54] Schmidt W (2001). Presentation accuracy of Web animation methods. Behav Res Methods.

